# New Horizons in Myeloproliferative Neoplasms Treatment: A Review of Current and Future Therapeutic Options

**DOI:** 10.3390/medicina57111181

**Published:** 2021-10-31

**Authors:** Domenico Penna

**Affiliations:** 1 Hematology Unit, Azienda Unità Sanitaria Locale—IRCCS, 42123 Reggio Emilia, Italy; domenico.penna@ausl.re.it; Tel.: +39-522-296-623; 2 Ph.D. Program in Clinical and Experimental Medicine, University of Modena and Reggio Emilia, 42121 Modena, Italy

**Keywords:** essential thrombocythemia, myelofibrosis, myeloproliferative neoplasms, Polycythemia vera, treatment

## Abstract

Philadelphia-negative myeloproliferative neoplasms (MPN) are aggressive diseases characterized by clonal proliferation of myeloid stem cells. The clonal process leads to excessive red cells production, platelets production, and bone marrow fibrosis. According to the phenotype, MPN can be classified as polycythemia vera (PV), essential thrombocythemia (ET), and primary myelofibrosis (PMF). MPN patients have shortened survival due to the increased risk of thrombosis, hemorrhages, and transformation to acute myeloid leukemia (AML). Prognosis is variable, with a shorter life expectancy in myelofibrosis. Currently, drug therapy can reduce symptoms, splenomegaly, and risk of thrombosis. Still, some patients can be resistant or intolerant to the treatment. At the same time, allogeneic stem cell transplant (ASCT) is the only treatment modality with the potential to cure the disease. Nevertheless, the ASCT is reserved for high-risk leukemic progression patients due to the risk of treatment-related death and comorbidity. Therefore, there is a need for new drugs that can eradicate clonal hematopoiesis and prevent progression to more aggressive myeloid neoplasms. Thanks to the better understanding of the disease’s molecular pathogenesis, many new potentially disease-modifying drugs have been developed and are currently in clinical trials. This review explores the most promising new drugs currently in clinical trials.

## 1. Introduction

Philadelphia-negative myeloproliferative neoplasms (MPN) are rare disorders characterized by clonal proliferation of myeloid stem cells. The myeloproliferation is induced by the so-called driver-mutations involving the genes of janus kinase 2 (*JAK2*), calreticulin (*CALR*), and myeloproliferative leukemia virus oncogene (*MPL*). Other molecular lesions (non-driver-mutations) are involved in the progression of the disease. This clonal process leads to excessive red cells production, platelets production, and bone marrow fibrosis. According to the phenotype, MPN can be classified as polycythemia vera (PV), essential thrombocythemia (ET), and primary myelofibrosis (PMF) [1]. MPN patients have shortened survival due to the increased risk of thrombosis, hemorrhages, and transformation to acute myeloid leukemia (AML) [2]. Furthermore, PV and ET can also evolve into secondary myelofibrosis (SMF). Prognosis is variable, with a shorter life expectancy in myelofibrosis (MF) than in PV and ET in both cases of PMF and SMF.

Furthermore, quality of life can be impaired by the presence of splenomegaly and constitutional symptoms [3,4]. Treatment decisions for MPN patients are influenced by the MPN subtype, symptom burden, and risk classification. Currently, drug therapy can reduce symptoms, splenomegaly, and risk of thrombosis. However, some patients can be resistant or intolerant to the treatment. At the same time, allogeneic stem cell transplant (ASCT) is the only treatment modality with the potential to cure the disease. Nevertheless, the ASCT is reserved for high-risk leukemic progression patients due to the risk of treatment-related death and comorbidity [5,6]. Therefore, there is a need for new drugs that can eradicate clonal hematopoiesis and prevent progression to more aggressive myeloid neoplasms. Thanks to the better understanding of the disease’s molecular pathogenesis, many new potentially disease-modifying drugs have been developed and are currently in clinical trials.

## 2. Molecular Biology

In the last 15 years, multiple somatic mutations have been associated with MPNs. In 2005, by identifying the *JAK2 V617F* mutation in most patients with PV, ET, or PMF, it was possible to start to understand the pathogenesis of this group of diseases [7,8,9]. Since then, many new molecular lesions have been found, and our knowledge of MPN biology has profoundly improved. Currently, we divide MPN mutations into two main groups: driver-mutations and non-driver-mutations. The first group includes mutations that activate the JAK-STAT pathway, leading to the disease phenotype [10,11]. The second group comprises many molecular lesions that affect genes involved in epigenetic regulation, messenger RNA splicing, transcriptional mechanisms, and signal transduction.

The mutations capable of activating the JAK-STAT pathway inducing the diseases’ phenotypic features involve three genes: *JAK2*, *CALR*, and *MPL*. The *JAK2 V617F* mutation results from somatic guanine to thymine mutation at nucleotide 1849 in exon 14 of the *JAK2* gene, leading to a single amino acid substitution from valine phenylalanine in codon 617 [10]. It is the most frequent driver mutation in MPN and is responsible for 95% of PV and approximately 50% of ET and PMF [7,12,13]. The remaining 5% of patients with PV JAK2 V617F negative harbor almost entirely mutations in *JAK2 exon 12* [14]. In contrast, most ET and PMF negative JAK2 V617F patients have detectable mutations in *MPL* or *CALR* [13,15,16,17], and only a minority have no detectable mutations (triple-negative). *MPL* mutations at tryptophan W515, located at the border of the transmembrane and cytosolic domains of MPL, are present in 3% of ET and 5% of PMF, with the most frequent mutations being W515L and W515K [16]. The most frequent *CALR* mutations are type 1, a 52 bp deletion, and type 2, a five bp insertion [17,18].

The second group of mutations (non-driver mutations) is not MPN specific, but it is relevant because it is associated with an increased risk of disease progression and shortened survival [5,19,20,21]. These numerous molecular lesions, in fact, often predict leukemic transformation and typically occur in the blast phase of the disease. It is possible to organize these kinds of mutations into three classes according to the gene function. The first one enlists mutations of genes involved in epigenetic regulation: *TET2*, *DNMT3A*, *IDH1/2*, *EZH2*, and *ASXL1* [10,22,23,24,25]. The second one comprises mutations in the RNA spliceosome machinery components, including *SF3B1*, *SRSF2*, *U2AF1*, and *SRSR2* [26,27]. The last involves mutations in transcription factors and signal transduction genes, enlisting *TP53*, *RUNX1*, *NRAS*, *SH2B3*, *CBL*, *NF1*, and *FLT3* [10,28,29,30,31].

## 3. The Clinical Course of the Disease

Despite the common genetic background, the disease phenotype and the clinical implications of the three conditions are different. In PV and ET, the excessive red blood cells and platelets are the diseases’ hallmark, while PMF is characterized by bone marrow fibrosis and cytopenias. Furthermore, all three clinical entities are accompanied by different degrees of cytokines deregulation, leading patients to develop splenomegaly and constitutional symptoms. These features explain the different clinical courses of the diseases.

Although, in comparison with the healthy population, MPN patients have greater chances of developing thrombosis (arterial and venous) and acute myeloid leukemia, these risks differ profoundly between the distinct clinical entities [32,33]. While thrombotic accidents are more common in PV and ET, leukemic progression prevails in PMF [34,35]. Therefore, it is crucial to develop tools able to predict the specific risk of each patient.

To this end, highly effective prognostic risk scores have been developed in the last ten years. On one side, thanks to a series of clinical and hematological parameters, it is now possible to predict the risk of thrombosis in PV and ET [36,37]. On the other, thanks to the new molecular acquisitions, it is possible to identify PMF patients at high risk of leukemic progression with increasing precision [38,39,40,41].

According to the patient-specific risk, the current therapeutic approach aims mainly to prevent thrombosis, reduce the risk of leukemic progression, and improve the quality of life by reducing spleen size, constitutional symptoms, and cytopenias. Standard treatment to prevent thrombosis involves using antiplatelet agents, anticoagulants, therapeutic venesections, and cytoreductive therapies [42,43,44]. Regarding the risk of leukemic evolution, ASCT offers the only treatment opportunity with the potential to cure the disease. However, the procedure is burdened by significant toxicity and a non-negligible risk of mortality in this population. Therefore, this option is generally reserved for patients at high risk of leukemic progression, with good performance status and longer life expectancy (in whom the procedure’s benefits outweigh the risks) [45,46].

## 4. Ruxolitinib Revolution

If 2005, with the discovery of the *JAK2* mutation, marked the beginning of the understanding of MPN molecular pathogenesis, then 2011, with the advent of ruxolitinib, was the starting year of target therapy in this setting [7,8,12,47,48]. Ruxolitinib is a potent and selective oral inhibitor of JAK1 and JAK2 and has been approved to treat MF and hydroxyurea-resistant or -intolerant PV. After ten years of using this JAK inhibitor in clinical practice and the execution of numerous clinical trials, it is now possible to highlight the advantages and limitations of the treatment. The main clinical benefits observed during therapy are SVR reduction and the improvement of the symptoms. Other significant benefits documented during the PMF clinical trials are an advantage in overall survival advantage (OS), as testified by the 5-year follow-up of the COMFORT studies, and an improvement in medullary fibrosis and allelic load in a minor part of patients, as shown in the COMFORT-2 study [49,50,51].

However, contextually to these advantages, the limits of the drug have also gradually emerged. The main clinical limitations are the onset of anemia and thrombocytopenia, especially at the start of treatment. Other significant problems related to the Ruxolitinib chronic use are the increased risk of opportunistic infections, skin cancer, and second malignant tumors [52,53,54,55,56,57].

## 5. New Therapeutic Options

Recently, many promising drugs have been developed to grant a more prolonged OS, overcoming ruxolitinib limitations. Thanks to the most recent molecular acquisitions, it has been possible to produce new agents capable of reducing thrombotic accidents, progression to leukemia, symptoms, and spleen size. Here, we report the most promising drugs currently in clinical trials. As shown in Table 1, these new therapeutic options are divided into three main groups:

1. new JAK inhibitors;
2. combination therapies;
3. non-JAK inhibitors new monotherapies.

### 5.1. New JAK Inhibitors

Four new JAK inhibitors, capable of overcoming ruxolitinib limitations providing different clinical benefits, have emerged in the last years: Momelotinib, Pacritinib, Fedratinib, and Jaktinib.

#### 5.1.1. Momelotinib—An Option for Anemic Patients with MF

Disease-induced or treatment-induced anemia is one of the most common problems in MF patients and significantly impacts OS. Momelotinib is a JAK and type 1 activin receptor inhibitor. Inhibition of the latter receptor downregulates hepcidin production in the liver, increasing iron and hemoglobin levels [58]. This mechanism of action was not known in the first clinical trials (SIMPLIFY-1 and SIMPLIFY-2), which did not include anemia reduction as a primary endpoint. In the SIMPLIFY-1 study, which involved JAK inhibitor treatment-naïve patients with MF, momelotinib demonstrated non-inferiority to ruxolitinib in reducing splenic volume (SVR), but not in reducing total symptoms score (TSS) [59]. The SIMPLIFY-2 trial compared momelotinib with the best available therapy (BAT) in patients previously exposed to ruxolitinib, documenting a significant reduction in TSS, but failing the primary endpoint of SVR [60]. Although, in both studies, the anemia-related endpoints favored momelotinib, the study design precluded formal statistical testing. Based on the favorable effect on anemia, Momelotinib is currently being studied in the MOMENTUM trial, which compares its efficacy to the use of danazol in patients pretreated with ruxolitinib.

#### 5.1.2. Pacritinib—An Option for Thrombocytopenic Patients with MF

Disease-induced or treatment-induced thrombocytopenia is another significant problem as well as an adverse prognostic factor in MF patients. Pacritinib, a relatively non-myelosuppressive inhibitor of JAK2 and FLT3 kinases, is proposed as a viable option in this patient subset. The main benefits observed in the PERSIST-1 clinical trial, which compared pacritinib and BAT (excluding ruxolitinib) in JAK inhibitor naïve patients, included improved hemoglobin and platelet levels and reduced SVR [61]. In the PERSIST-2 study, which involved patients with MF and thrombocytopenia (platelets < 100 × 109/L), an attempt was made to identify the dose associated with the most significant benefit; the patients were randomized to two doses of pacritinib (200 mg BID or 400 mg once daily) or BAT (including ruxolitinib). Pacritinib, especially at a dose of 200 mg twice daily, was superior to BAT, especially in SVR at 24 weeks. A further study was then conducted to assess cardiac and hemorrhagic risk: PAC203 [62]. The 200 mg twice daily dose was well tolerated and emerged as a winner. Currently, the Phase III PACIFICA registration study is evaluating the safety and efficacy of pacritinib 200 mg BID versus physician’s choice in patients with MF and severe thrombocytopenia (<50 × 109/L) and less than 12 weeks of previous therapy with JAK inhibitors [63].

#### 5.1.3. Fedratinib—An Option for Patients with MF Resistant or with Sub-Optimal Response to JAK Inhibtors

Some patients with MF may be resistant, intolerant, ineligible, or lose response to Ruxolitinib. Fedratinib, a JAK and FLT3 inhibitor, already approved in the US but not yet in Europe, is the first active drug in this group of patients. FDA approval was granted based on Phase II and the Phase III clinical trials JAKARTA. These randomized, placebo-controlled trials have shown a significant reduction in TSS and SVR in patients treated with fedratinib [64,65,66,67]. Despite these results, the development of this new JAK inhibitor was problematic due to concerns about Wernicke encephalopathy [65].

For this reason, not only have JAKARTA studies been suspended prematurely, but the JAKARTA-2 trial was permanently discontinued due to the loss of week 24 data in many patients. Although encephalopathy occurred in only 1% of patients, and most cases were not Wernicke’s encephalopathy, fedratinib has a black box warning for this complication [68]. To gain more experience and integrate the missing information from the JAKARTA studies, currently, fedratinib is being studied in the FREEDOM trials in the post-ruxolitinib setting (alone or in comparison with BAT).

#### 5.1.4. Jaktinib—An Option for Patients with MF Resistant or with Sub-Optimal Response to JAK Inhibtors

Jaktinib is a new promising inhibitor of JAK1 and JAK2 kinases. This drug is currently being studied in the Phase 2 multicenter trial ZGJAK002 [69]. This study involves patients with intermediate-to-high risk MF (primary or secondary) evaluating two different dosing regimens: 100 mg BID or 200 mg QD. Preliminary results seem to favor the 100 mg BID arm regarding SVR, while they are comparable in terms of TSS and reduction of transfusion dependence. Furthermore, with both posologies, the drug is generally well-tolerated and safe in MF patients.

### 5.2. Combination Therapies

Unlike the new JAK inhibitors, combination therapies aim not to replace ruxolitinib, but to increase its efficacy and improve its tolerability by acting on other molecular targets. Many combinations have shown synergism in the preclinical setting; however, not all treatments have proven effective in clinical practice. The most promising drugs currently included in clinical trials are Luspatercept, Parsaclisib, Navitoclax, and Pelabresib.

#### 5.2.1. Luspatercept—An Option for Anemic Patients with MF

One of the main limitations in the use of Ruxolitinib is anemia. Therefore, the use of anemia therapy in combination with the JAK inhibitor is an effective strategy to improve the tolerability of the drug. Luspatercept is an erythroid maturation agent that acts as an activin receptor ligand and is currently approved for treating anemia in myelodysplastic syndrome [70,71]. A Phase 2 clinical trial analyzed the association between ruxolitinib and luspatercept, administered subcutaneously every 21 days, showing encouraging results [72]. The drug was, in fact, able to significantly increase hemoglobin levels and reduce the rate of blood pressure transfusion in treated patients. For this reason, a Phase 3 study is planned, which confirms the preliminary results and better defines the safety profile of the drug.

#### 5.2.2. Parsaclisib—An Option for Patients with MF Resistant or with Sub-Optimal Response to JAK Inhibitors

A part of the patients treated with ruxolitinib shows only a partial response to the treatment. In this setting, a complementary therapy able to enhance the action of the JAK inhibitor could allow patients to reach a complete response. Parsaclisib, a PI3Kδ inhibitor, was studied in a Phase 2 clinical trial combined with ruxolitinib in patients with a suboptimal response (palpable spleen ≥ 10 cm or 5–10 cm, with active symptoms) after at least six months of treatment with the JAK inhibitor [73]. The study compared two dosing schedules: 1—QD for eight weeks followed by one dose weekly; 2—daily. The daily dosing was associated with greater efficacy in terms of both SVR and TSS. Furthermore, the drug also was well tolerated with limited grade 3 and 4 adverse events. For this reason, Parsaclisib (at the daily dosage) in combination with ruxolitinib will be soon evaluated in a Phase 3 study [74].

#### 5.2.3. Navitoclax—An Option for Patients with MF Resistant or with Sub-Optimal Response to JAK Inhibitors

Preventing or reversing resistance to ruxolitinib is a primary therapeutic goal of combination therapies. To this end, a Phase 2 trial recently evaluated Navitoclax, a non-selective inhibitor of Bcl2, in combination with ruxolitinib. The drug’s apoptotic effect improved response to ruxolitinib in highly treatment-resistant MF patients. In particular, the study enrolled patients who had received ≥12 weeks of continuous ruxolitinib therapy and had persistent splenomegaly. The combination of navitoclax with ruxolitinib was generally well-tolerated and resulted in significant SVR, improvement in TSS, and reduction in bone marrow fibrosis (BMF) and cytokine deregulation. The results are particularly encouraging given the efficacy of the treatment in a population usually characterized by limited therapeutic options and often by low life expectancy. Therefore, navitoclax and ruxolitinib will now be evaluated in a randomized Phase 3 trial in treatment-naïve patients [75].

#### 5.2.4. Pelabresib—An Option for Patients with MF Resistant or with Sub-Optimal Response to JAK Inhibitors

Another promising candidate to overcome ruxolitinib resistance is Pelabresib, both as a combination therapy and as a single agent. Pelabresib is a bromodomain and extraterminal domain inhibitor, which modulates NFκB and TGF-β signaling pathways [76]. The MANIFEST study used a two-arm design, one arm consisting of Pelabresib monotherapy in patients who are refractory/intolerant to ruxolitinib, and the other arm consisting of Pelabresib in combination with ruxolitinib; significant reduction of SVR and TSS were highlighted [77]. In addition, improvements in anemia and BMF levels were reported, and treatment was well tolerated. Based on these encouraging results, a Phase 3, double-blind, randomized study was initiated comparing the combination CPI-0610 and ruxolitinib with ruxolitinib alone (MANIFEST-2).

### 5.3. Non-JAK Inhibitors New Monotherapies

Some MF patients could lose the clinical response, be resistant, or be ineligible for therapy with JAK inhibitors. Furthermore, in PV and ET settings, at the moment, the therapeutic options are also more limited. Therefore, many non-JAK inhibitors single agents are currently investigated in clinical trials. The five most intriguing novel drugs are Imetelstat, KRT232, Bomedemstat, Rusfertide, Besremi, and Givinostat.

#### 5.3.1. Imetelstat—An Option for Patients with MF Refractory or Ineligible for JAK Inhibitors

Treatment options alternative to JAK inhibitors could be essential in patients ineligible for or refractory to these drugs. Imetelstat is a competitive inhibitor of the telomerase enzyme complex with promising activity in patients with JAK inhibitor-resistant MF [78]. The Phase 2 IMBARK study recently evaluated two dose levels of imetelstat (4.7 mg/kg and 9.4 mg/kg) administered intravenously every three weeks. The study involved intermediate/high-risk MF patients who failed therapy with a JAK inhibitor (no reduction in splenomegaly after 12 weeks or worsening splenomegaly). While the lower dose arm did not show sufficient drug activity, the 9.4 mg/kg arm was associated with response in terms of SVR and TSS and a significant advantage in OS [79]. Given these encouraging results, the imetelstat Phase 3 registration study has been initiated, with the advantage of OS as the primary endpoint.

#### 5.3.2. KRT232—An Option for Patients with MF Refractory or Ineligible for JAK Inhibitors

As previously seen, *TP53* mutations are rare in MPN in the chronic phase, while they are often present in the blast phase [3,19,28,30]. However, p53 may still be inhibited due to the JAK2 V617F mutation, capable of increasing the expression of MDM2, the physiological inhibitor of p53 [80]. KRT-232, an MDM2 inhibitor, has been evaluated in a Phase 2 trial of MF patients with non-mutated TP53 but who have relapsed after or are refractory to JAK inhibitor therapy [81]. The study did not include patients who are intolerant to JAK inhibitor therapy or severely thrombocytopenic (platelet counts below 50 × 109/L). Of the four therapeutic regimens studied, 240 mg/day on days 1–7 every four weeks was associated with greater efficacy in terms of SVR and TSS and good tolerability. For these reasons, the randomized Phase 3 trial was recently launched, comparing BAT with KRT-232 at a dosage of 240 mg/d on days 1–7 every four weeks.

#### 5.3.3. Bomedemstat—An Option for Patients with MF or ET with Thrombocytosis

Lysine-specific demethylase 1 (LSD1) is an overexpressed epigenetic enzyme in MPN, which promotes erythropoiesis, granulopoiesis, thrombopoiesis, inflammation, and fibrosis [82]. The LSD1 inhibitor bomedemstat was analyzed in a Phase 2 study in patients with intermediate-2/high-risk MF resistant or intolerant to ruxolitinib [83]. A minimum baseline platelet count of 100 × 109/L was required, and considering the expected thrombocytopenia, the drug’s dosage was individualized to achieve a target platelet count of 50 × 109/L. No dose-limiting toxicities or maximum tolerated dose were identified. The drug was able to improve TSS and reduce SVR. In addition, improvements were observed in BMF, anemia, and the frequencies of mutant alleles. Given the results and considering the thrombocytopenic effect of the drug, bomedemstat is now an object of study not only in the MF setting, but also in the ET setting for patients who have failed at least one standard therapy.

#### 5.3.4. Rusfertide—An Option for Patients with PV Needing Phlebotomy

Patients with PV require periodic phlebotomies to keep hematocrit levels <45%, but between procedures, hematocrit levels may rise above this threshold, and patients may be at risk for thrombosis. Additionally, periodic phlebotomies can cause systemic iron deficiency, often associated with thrombocytosis, further increasing thrombotic risk. Hepcidin is a negative regulator of iron metabolism, capable of reducing the availability of iron and the need for phlebotomies. Rusfertide is a hepcidin mimetic currently studied in a Phase 2 study, comparing iron status and phlebotomy requirements before and during drug treatment in patients with PV and a high phlebotomy need. Rusfertide doses of 10, 20, 40, 60, and 80 mg administered subcutaneously weekly were added to each subject’s treatment for PV, and the dose was adjusted to maintain hematocrit <45%. The drug was well-tolerated, and patients’ need for phlebotomy decreased significantly during treatment, while ferritin levels gradually approached normality [84]. These results indicate that rusfertide is an effective agent for the treatment of PV, reversing iron deficiency and eliminating the need for phlebotomies in patients with PV.

#### 5.3.5. Besremi—An Option for Patients with PV Needing Cytoreduction

Ropeginterferon alfa-2b is a new mono-pegylated interferon that can be administered every two weeks. The drug, called Besremi, is being studied within the PROUD-PV/CONTINUATION-PV Phase III clinical trial [85]. In the first part of the study (PROUD-PV), patients who were treatment-naïve or who had been treated with HU <3 years without achieving a CHR were randomized to HU against Besremi. Once the primary endpoint of non-inferiority in TSS and SVR was reached, the study proceeded with the second part (CONTINUATION-PV). Patients in the Besremi arm continued with the same therapy, while patients enrolled in the HU arm were treated with BAT (which included HU, pegIFNα-2a, and JAK inhibitor). Not only was the hematological response (CHR) significantly higher in the Besremi arm, but there was also a more profound molecular response characterized by a significant decrease in the JAKV617F VAF. Recent real-world data seem to confirm Besremi efficacy and safety in the treatment of MPN [86].

#### 5.3.6. Givinostat—An Option for Patients with PV Needing Cytoreduction

Histone deacetylase (HDAC) is an enzyme that exerts epigenetic control by reducing the expression of tumor suppressor genes. However, this oncogenic activity can be counteracted by HDAC inhibitors. Givinostat is a drug belonging to this new group of inhibitors with the ability to prevent the synthesis of the mutated JAK2 protein [87]. For this reason, its use in the context of PV has been investigated. Studies have shown benefits in both complete and partial hematological recovery, improvement of splenomegaly, and reduction of itching. In addition, a reduction in allelic load has also been documented in some patients [88]. The drug was also analyzed in combination with HU, showing a synergistic effect between the two drugs, with better control of symptoms and hematocrit values [89]. Therefore, this new HDAC inhibitor could represent an intriguing therapeutic option for patients with PV resistant to standard therapy.

## 6. Conclusions

Treatment options for MPN have changed dramatically over the past decade. Recent molecular acquisitions in this area have allowed the development of various therapeutic options capable of overcoming the limits of standard treatment. Although ABMT remains the only treatment option at the moment, new therapeutic agents have the potential to alter the natural history of the disease; reduce the risk of leukemic transformation, thrombotic accidents, cytopenia; and improve survival and quality of life in patients with MPN.

**Figure 1:**
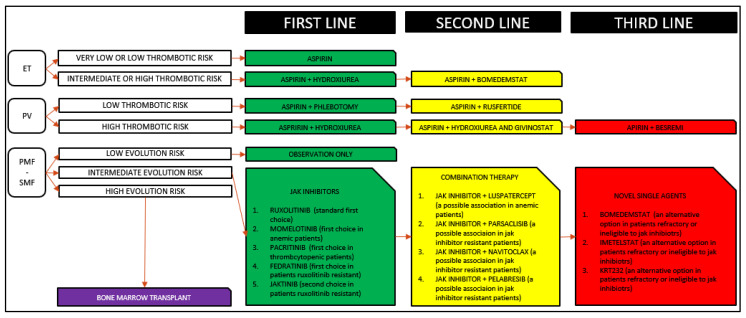
Reported at the end of the manuscript.

Soon, the space dedicated to nonspecific cytoreductive therapies will decrease, while JAK inhibitors will probably progressively acquire more importance, becoming the cornerstone of MPN treatment. In this setting, however, ruxolitinib will not be the only clinical option, but it will be possible to choose a different JAK inhibitor according to the patient’s specific needs (anemia, thrombocytopenia, previous exposure to another JAK inhibitor). Furthermore, in case of resistance or suboptimal response to treatment, it will be possible to introduce combination therapies, while in case of intolerance, it will be possible to use new alternative drugs to JAK inhibitors. In this way, not only will clonal progression towards more aggressive forms of the disease be reduced, but thrombotic events will also be controlled more effectively, improving OS (Figure 1).

However, with all these therapeutic options available, it will soon be necessary to dispose of accurate predictors of response to treatment and precise response criteria to therapies. Many studies are expected to identify the new molecular markers’ prognostic, predictive, and therapeutic roles. This research will have a massive impact as it will allow the choice of therapy and control of the course of the disease. Furthermore, it is reasonable to assume that information about the potential and limitations of new drugs will also increase. These two conditions will allow the researchers to create a systematic treatment approach based on the patient’s specific characteristics.

## Figures and Tables

**Table 1:** Reported at the end of the manuscript: 5.1 New JAK inhibitors.

Treatment Strategy	Drug	Mechanism of Action	Phase—NCT Number (Trial)	Study Design	Status
Jak inhibitors	Momelotinib	JAK1-JAK2-ACVR1 inhibitor	3—NCT01969838 (SIMPLIFY 1)	Momelotinib vs. Ruxolitinib in PMF or SMF	Completed
			3—NCT02101268 (SIMPLIFY 2)	Momelotinib vs. BAT in anemic or thrombocytopenic PMF or SMF	Completed
			3—NCT04173494 (MOMENTUM)	Momelotinib vs. Danazol in anemic PMF or SMF	Ongoing
	Pacritinib	JAK2-FLT3-IRAK1-CSF1R inhibitor	3—NCT01773187 (PERSIST 1)	Pacritinib vs. BAT in PMF or SMF	Completed
			3—NCT02055781 (PERSIST 2)	Pacritinib vs. BAT in PMF or SMF and thrombocytopenia	Completed
			3—NCT03165734 (PACIFICA)	Pacritinib vs. BAT in PMF or SMF and severe thrombocytopenia	Ongoing
	Fedratinib	JAK2-FLT3 inhibitor	3—NCT01437787 (JAKARTA 1)	Fedratinib vs. placebo in PMF or SMF	Completed
			2—NCT01523171 (JAKARTA 2)	Fedratinib in PMF or SMF previously treated with Ruxolitinib	Completed
			3—NCT03755518 (FREEDOM 1)	Fedratinib in PMF or SMF previously treated with Ruxolitinib	Ongoing
			3—NCT03952039 (FREEDOM 2)	Fedratinib vs. BAT in PMF or SMF previously treated with Ruxolitinib	Ongoing
	Jaktinib	JAK1-JAK2-JAK3 inhibitor	2—NCT03886415	Jaktinib in PMF or SMF	Ongoing
Combination therapies with ruxolitinib	Luspatercept	Activin ligand trap	2—NCT03194542	Luspatercept +/− Ruxolitinib in PMF	Ongoing
	Parsaclisib	PI3Kδ inhibitor	2—NCT02718300	Parsaclisib + Ruxolitinib in PMF or SMF	Ongoing
	Navitoclax	BCL2/BCL-Xl inhibitor	2—NCT03222609 (REFINE)	Navitoclax + Ruxolitinib in PMF or SMF	Ongoing
	Pelabresib	BET inhibitor	2—NCT02158858 (MANIFEST)	Pelabresib +/− Ruxolitinib in PMF or SMF	Ongoing
			3—NCT04603495 (MANIFEST 2)	Pelabresib + Ruxolitinib vs. Placebo + Ruxolitinib in PMF or SMF	Ongoing
Novel agents	Imetelstat	Telomerase inhibitor	2—NCT02426086 (IMBARK)	Imetelstat in PMF previously treated with Ruxolitinib	Completed
	KRT232	MDM2 inhibitor	2—NCT03662126 (BOREAS)	KRT232 in PMF or SMF previously treated with Ruxolitinib	Ongoing
			3—NCT03662126 (BOREAS)	KRT232 vs. BAT in PMF or SMF previously treated with Ruxolitinib	Ongoing
	Bomedemstat	LSD1 inhibitor	2—NCT03136185	Bomedemstat in PMF or SMF	Ongoing
			2—NCT04254978	Bomedemstat in ET	Ongoing
	Rusfertide	Hepcidin mimetic	2—NCT04057040	Rusfertide in PV	Ongoing
	Besremi	Interferon-α	3—NCT02218047 (CONTI-PV)	PEG-P-INF alpha-2b vs. BAT in PV	Completed
	Givinostat	Histone deacetylase inhibitor	2—NCT0060307	Givinostat in MPN	Completed
			2—NCT00928707	Givinostat + HU in MPN	Completed
